# Valedictory

**Published:** 1866-01

**Authors:** 


### EDITORIAL.

### VALEDICTORY.

With the present number we transfer the editorial and business management of the JOURNAL to other hands. That the past two years have been years of unusual difficulty for medical periodicals, is sufficiently indicated by the number that have been discontinued during this time; yet we have never had occasion for discouragement or complaint. In this period our subscription list has received a constant and healthful increase, while our patrons have met their pecuniary obligations so promptly and universally as to obviate every trouble of this nature. True, there are some exceptions to this praiseworthy conduct, but they are less numerous than we had reason to expect. The only embarrassment we have experienced has come from lack of time to give to the pages of the JOURNAL the labor that justice to our subscribers demanded; and year by year this difficulty is increased by the press of duties from which we cannot escape. This consideration has induced us to place the JOURNAL in charge of those who will give it all the attention it requires. In an editorial and business intercourse of a number of years with its readers, scarcely a circumstance has occurred of an unpleasant nature, which allows the mind to dwell with satisfaction upon this period of our labor. From our correspondence, we feel personally acquainted with many whom we have never met. We can only express our thanks to those who have aided us in our work, and favored our readers by scientific contributions to the pages of the JOURNAL, and to bespeak for those who assume our duties the same kindness and forbearance that we have received.

EX-EDITORS.

